# Editorial: The endocrine role of the musculoskeletal system

**DOI:** 10.3389/fendo.2025.1552950

**Published:** 2025-01-23

**Authors:** Siyi Zhu, Giuseppina Storlino, Sandeep Kumar

**Affiliations:** ^1^ Rehabilitation Medicine Center and Institute of Rehabilitation Medicine, West China Hospital, Sichuan University, Chengdu, China; ^2^ Key Laboratory of Rehabilitation Medicine in Sichuan Province, West China Hospital, Sichuan University, Chengdu, China; ^3^ Department of Clinical and Experimental Medicine, University of Foggia, Foggia, Italy; ^4^ Department of Genetics, University of Alabama at Birmingham, Birmingham, AL, United States

**Keywords:** musculoskeletal endocrinology, bone-muscle crosstalk, myokines and osteokines, systemic homeostasis, metabolic bone diseases

The musculoskeletal system’s dual role as a structural framework and an endocrine organ has unveiled a new frontier in understanding its systemic influence (1–3). This Research Topic focuses on the endocrine crosstalk between bone and muscle, emphasizing the production and regulation of myokines and osteokines, and their roles in maintaining systemic homeostasis (2–5). The collected studies explore how the musculoskeletal system contributes to both physiological and pathological conditions, enriching the field of endocrinology with innovative insights.

## MicroRNAs in bone loss and diabetes

Daamouch et al. examine the role of microRNAs in Type 1 diabetes-induced bone loss, identifying key dysregulated miRNAs in serum and bone tissues. This study highlights miR-136-3p and miR-206-3p as pivotal biomarkers and their links to pathways like TGF-beta and osteoclast differentiation, advancing potential diagnostics for bone fragility.

## Vitamin B12’s role in musculoskeletal health

Zhao et al. investigate the impact of Vitamin B12 and its biomarkers on musculoskeletal health, revealing significant associations with bone mineral density (BMD) and muscle strength in older adults. These findings underscore the potential of Vitamin B12 as a critical determinant of aging-related musculoskeletal integrity.

## Sclerostin levels and metabolic disorders

Alramah et al. analyze sclerostin levels in a multiethnic population with obesity and Type 2 diabetes, uncovering significant gender and ethnic differences. Elevated sclerostin correlated with metabolic markers and bone health, emphasizing its role as a potential biomarker for metabolic bone diseases.

## Glucocorticoid receptor in bone marrow adipocytes

Schill et al. investigate the effects of glucocorticoid receptor deficiency in bone marrow adipocytes, revealing mild impacts on bone and hematopoiesis but no influence on marrow adiposity expansion under caloric restriction. This study adds nuance to our understanding of marrow adipose tissue’s endocrine function.

## Fatty acids and adolescent bone health

Wang et al. link dietary fatty acids to bone mineral density in adolescents, finding that saturated fatty acids enhance BMD while polyunsaturated fats have a negative effect. This study highlights the importance of dietary balance in adolescent skeletal development.

## Lead exposure and bone density

Wang et al. reveal a significant negative correlation between urinary lead levels and BMD, emphasizing the toxicological impact of environmental lead on bone health. This study calls for heightened public health initiatives targeting heavy metal exposure.

## Sarcopenia and rotator cuff tears

Yang et al. establish a genetic link between sarcopenia-related traits and rotator cuff tears using Mendelian randomization, providing evidence-based insights for optimizing clinical management of these conditions.

## FGF21 and osteoporosis

Liu et al. explore the causal effects of FGF21 overexpression on bone health, demonstrating its role in reducing BMD and increasing osteoporosis risk. This study identifies FGF21 as a potential therapeutic target for bone-related metabolic disorders.

## Vascular and lymphatic networks in bone health

Huang et al. review the interplay between vascular and lymphatic systems in bone and joint homeostasis, highlighting their co-regulatory roles and potential therapeutic implications for inflammatory joint diseases.

## Acupuncture and lumbar disc herniation

Yan et al. compare acupuncture to traditional rehabilitation in lumbar disc herniation, finding superior long-term benefits in muscle restoration and pain relief. This research underscores acupuncture’s potential as an integrative therapy.

## Conclusion

Collectively, these studies enhance our understanding of the musculoskeletal system’s endocrine roles and its systemic interactions. We thank the authors for their valuable contributions, advancing both fundamental and clinical endocrinology. Their work lays a robust foundation for future interdisciplinary research.

## References

[1] DiGirolamo DJ Clemens TL Kousteni S. The skeleton as an endocrine organ. Nat Rev Rheumatol. (2012) 8:674–83. doi: 10.1038/nrrheum.2012.157 23045255

[2] Van Gastel N Carmeliet G. Metabolic regulation of skeletal cell fate and function in physiology and disease. Nat Metab. (2021) 3:11–20. doi: 10.1038/s42255-020-00321-3 33398192

[3] Zhou R Guo Q Xiao Y Guo Q Huang Y Li C. Endocrine role of bone in the regulation of energy metabolism. Bone Res. (2021) 9:25. doi: 10.1038/s41413-021-00142-4 34016950 PMC8137703

[4] Gomarasca M Banfi G Lombardi G. Myokines: The endocrine coupling of skeletal muscle and bone. Adv Clin Chem. (2020) 94:155–218. doi: 10.1016/bs.acc.2019.07.010 31952571

[5] Wan M Gray-Gaillard EF Elisseeff JH. Cellular senescence in musculoskeletal homeostasis, diseases, and regeneration. Bone Res. (2021) 9:41. doi: 10.1038/s41413-021-00164-y 34508069 PMC8433460

